# ERMA: a harmonizing epicPCR data analysis tool

**DOI:** 10.1186/s12864-026-13418-y

**Published:** 2026-10-09

**Authors:** Adrian Dörr, Svjetlana Dekić Rozman, Lina Viktoria Duncker, Jan Kehrmann, Jan Buer, Marko Virta, Folker Meyer, Ivana Kraiselburd

**Affiliations:** 1 Data Science Research Group, Institute for Artificial Intelligence in Medicine (IKIM), University Hospital of Essen, University of Duisburg-Essen, Essen, Germany; 2 Institute of Microbiology, University of Helsinki, Helsinki, Finland; 3 Institute for Medical Microbiology, University Hospital Essen, Essen, Germany

**Keywords:** epicPCR, Data analysis, Workflow, Standardization

## Abstract

**Background:**

EpicPCR links functional genes to taxonomic markers at the single-cell level and has become an important method in microbial ecology and antimicrobial resistance research. However, the lack of missing standardized data analysis workflows limits reproducibility and comparability across studies. Existing analyses are often based on custom, unpublished scripts, complicating cross-study benchmarking and reuse.

**Results:**

We present ERMA, an open-source, Snakemake-based pipeline that enables an automated, standardized, reproducible, and scalable epicPCR data analysis with minimal user input. ERMA supports both Illumina and ONT sequencing data and integrates quality control, database preparation, dual similarity searches against taxonomic and functional reference databases, results integration, filtering, and visualization. The pipeline is adaptable to a broad range of research questions beyond AMR due to modular database preparation and integration of specific UniRef queries. We evaluated ERMA using five publicly available epicPCR datasets covering diverse sample sources and sequencing depths. Across these datasets, ERMA achieved mean concordance rates of approximately 70–83% with the taxonomic results reported in the respective publications. In addition, validation using a defined five-member mock community spiked into clinical wastewater demonstrated full recovery of mock genera, with 85% of ERMA-detected genera also detected by independent 16S rDNA gene analysis of the same wastewater sample. Attrition analysis showed inconsistent filtering patterns across studies, reflecting dataset-specific quality differences.

**Conclusion:**

While large-scale reference datasets for epicPCR remain limited, validation using both publicly available studies and a defined mock community shows strong, interpretable performance across heterogeneous datasets. By providing a transparent, modular, and reproducible workflow, ERMA contributes toward harmonized epicPCR data analysis and establishes a foundation for future methodological standardization and benchmarking efforts as reference datasets improve.

## Background

Antimicrobial resistance (AMR) represents a major threat to global public health. An estimated 4.71 million deaths worldwide have been associated with bacterial AMR, with estimates showing an increase to 8.22 million deaths annually by 2050 [1]. The emergence and spread of AMR is driven by numerous factors, including inappropriate antimicrobial use, extensive application of antibiotics in veterinary medicine and insufficient access to clean water and food sources [2]. Addressing this complex problem has led to the establishment of the One Health framework, which integrates human, animal, and environmental health perspectives to better understand the AMR dynamics in interconnected ecosystems [3]. Within this framework, reliable, cost-efficient, and scalable surveillance methods are important.

A wide range of phenotypic and molecular methods is currently used for AMR detection. Traditional culture-based assays, although frequently labor-intensive and time-consuming, provide direct phenotypic confirmation of antimicrobial resistance. Automated phenotypic systems such as BD Phoenix and VITEK2 reduce turnaround times and increase throughput, although at a higher initial cost [4]. Molecular approaches offer the complementary advantage of cultivation-independent detection, providing access to non-culturable microorganisms. Quantitative PCR (qPCR) offers high sensitivity and specificity for targeted resistance genes but lacks contextual information regarding the host organism or the cellular origin of the detected DNA [5]. Similarly, 16S rDNA gene amplicon sequencing allows comprehensive profiling of microbial community composition but does not provide information on functional traits, such as antimicrobial resistance [6]. Shotgun metagenomics allows simultaneous taxonomic and functional profiling, including AMR genes (ARG), but remains costly, analytically complex, and limited in its ability to resolve low-abundance organisms [7]. For environmental samples, where AMR surveillance is especially valuable, a cost-efficient method is desired to connect taxonomic and functional signals.

EpicPCR (Emulsion, Paired Isolation and Concatenation PCR) approaches this gap by physically linking two genomic regions of interest within single cells, most commonly antimicrobial resistance genes and 16S rDNA gene fragments [8]. Using emulsion-based compartmentalization and a linker primer strategy, epicPCR generates chimeric fusion amplicons that preserve host-gene associations. This approach has been successfully applied in multiple studies to identify host taxa that carry specific resistance genes in complex microbial communities [9–13].

However, epicPCR data analysis presents considerable bioinformatic challenges. The resulting chimeric reads resemble artifacts that are typically removed in conventional sequencing analysis workflows. Hence, data analysis is often custom-built, unpublished, or adapted for individual studies, showing a lack of formalized algorithms and decision logic. Consequently, the reproducibility, comparability, and standardization across epicPCR studies remain limited.

To address these gaps, we developed ERMA (EpicPCR Resistome-Microbiome Analyzer), a version controlled, open-source, Snakemake-based [14] workflow designed to automate, standardize, and reproducibly analyze epicPCR data. ERMA v1.0.0 supports sequencing data from both Illumina and Oxford Nanopore Technologies (ONT) platforms and performs dual similarity searches against two user-defined reference databases, with CARD and SILVA as defaults for ARGs and 16S rDNA, respectively [15, 16]. By combining quality control, similarity searching, filtering, and visualization into a single automated pipeline, ERMA enables standardized analysis of epicPCR data and facilitates harmonized downstream interpretation. The workflow is open-source and is available on GitHub (https://github.com/IKIM-Essen/ERMA/).

To evaluate ERMA’s robustness and applicability, we reanalyzed publicly available epicPCR datasets from five independent studies [9–13] and compared the results with those originally reported. Given the heterogeneity of epicPCR studies and the absence of a known ground truth, this concordance analysis provides a relative comparison to assess consistency, reproducibility, and analytical concordance. Additionally, to approach controlled validation, we performed epicPCR on a mock community containing known resistances, which we spiked into a WW sample. We then analyzed the results using ERMA and compared the identified genera with those found in an independent 16S rDNA analysis of the same WW sample.

## Implementation

### Pipeline overview and design principles

ERMA is an open-source, Snakemake-based workflow [14] designed to automate and standardize epicPCR data analysis in a reproducible and scalable manner. The pipeline is built to focus on robustness, computational speed, and specificity. We chose Snakemake as the workflow-management system because its modular, rule-based architecture is well suited to integrating a wide variety of bioinformatics tools (Table 1) needed to analyze complex epicPCR data. Additionally, this approach provides code flexibility, allowing individual pipeline components to be modified, replaced, or extended without altering the overall workflow’s functionality. Using Conda [17] for dependency management provides stability by managing version control of the packages and tools used.

**Table 1:** Bioinformatic tools integrated in the pipeline with the respective steps and versions they are used in. References are shown at the end of the table

Stage	Step	Tool	Version	Reference
General workflow		Snakemake	>9.0	[14]
Pre-process	Merge Illumina paired-end reads	Ngmerge	0.3	[18]
	Demultiplex ONT input	Seqkit	2.10.0	[19]
	Prepare FASTQ	Seqtk	1.3	[20]
Quality control	Multiqc	FastQC wrapper	6.0.1	[21]
		Multiqc wrapper	6.0.1	[22]
Database	Preparing Database	CARD	4.0.1	[15]
		SILVA	138.2	[16]
	Optional Integrating	UniProt	2025_04	[23]
		UniRef		[24]
		UniProt API		[25]
Similarity Search	16S rDNA Similarity search	USEARCH	12.0 (beta)	[26]
	ARG/Target Similarity search	Diamond	2.1.10	[27]
Post-Process	Merge hits	Pandas	2.2.3	[28]
	Filter hits			
Plotting	Boxplots, HTML tables, Bubble, Stacked abundance	Seaborn	0.13.2	[29]
		Matplotlib	3.9.1	[30]
		Pandas	2.2.3	[28]
		Plotly	5.14.1	[31]
		Numpy	2.3.5	[32]
Output	Report	Snakemake	7.32	[14]

**Fig. 1:**
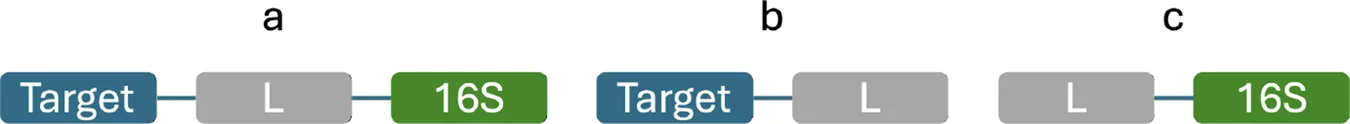
Schematic overview of possible epicPCR amplification outcomes. **a** Representation of a complete fusion between the ARG target and the 16S rDNA gene via the linker sequence. **b** and (**c**) Partial byproducts lacking either the 16S rDNA gene or the target region, respectively

**Fig. 2:**
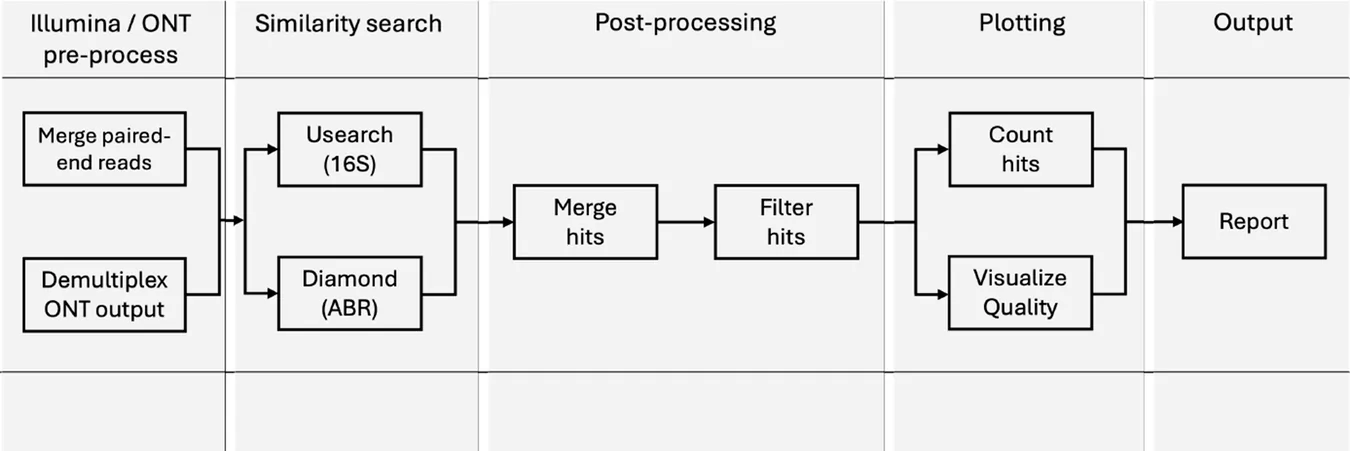
Sketch of the ERMA main functionalities: preprocessing, similarity search, postprocessing, plotting, and concatenating results in a report

EpicPCR data is chimeric by design: the desired fusion reads comprise the targeted region, often ARG, the linker primer, and the 16S rDNA gene region in one read (Fig. 1a). Amplification products containing only the target (Fig. 1b) or the 16S rDNA gene (Fig. 1c) with the linker are biologically not meaningful and will be removed throughout the analysis. This structure shaped the pipeline architecture: two independent local similarity searches are performed for the two parts of the complete fusion reads. The results of these dual searches are then merged and filtered during post-processing, plotted, and concatenated with other results in a single report (Fig. 2).

The pipeline structure supports input from any sequencing platform and has been specifically tested with Illumina and ONT data. Since the downstream filtering logic is mainly based on the percentage identity of the query-reference comparison, platform-based differences in read length are of secondary importance for the analysis. Specificity may be affected by platform-specific error profiles. However, the pipeline is designed to identify the optimal match, delivering the best possible analysis results across datasets with varying sequence quality.

### Preprocessing of Illumina or ONT inputs

ERMA provides a preprocessing module (data_prep) that converts raw sequencing outputs into a unified file format, specifically for Illumina and ONT sequencing. For this, the user must copy the input data to ERMA’s data/fastq folder and then start the module. The file names are automatically extracted and employed in the analysis results. For the main pipeline, the data need to be in a one-file-per-sample format. Hence, for Illumina paired-end reads, a merging module is incorporated. For ONT input, a similar module can be used to process the raw sequencing output. With an additional user-generated input file that links barcodes to sample names, the module automatically demultiplexes the reads and prepares the resulting files for the main analysis pipeline.

### Quality control

ERMA uses several modules to provide users with comprehensive quality control. FastQC [21] and MultiQC [22] are used to create an overall overview of input read quality. This generates a consolidated and standardized overview of sequence counts, read quality, per-base quality, length distribution, and related metrics. In addition, an overview table is created, providing information on quantities of intermediate results throughout the pipeline, namely the number of hits generated by the similarity search (Similarity search strategy section), the number of filtered and accepted hits at each filtering step, and the number of high-quality fusion reads (Result integration and filtering logic section). For visualization, this data is used to create an attrition plot (Visualization section).

Quality control is descriptive and supports user-driven sample exclusion: the attrition plot is particularly useful for distinguishing between high- and low-performing samples, while the MultiQC report offers a broad overview of sample quality.

### Database handling

To approach epicPCR data analysis based on chimeric reads, two different databases were used. By default and for the current version of the pipeline, these databases are CARD [15] version 4.0.1 for ARGs and SILVA [16] version 138.2 for 16S rDNA genes. These databases were chosen for their lightweight and comprehensiveness. In the default configuration, the databases are downloaded once throughout the pipeline, with links defined in the configuration file. However, the user can also select alternative versions or databases by modifying those links. It is also possible to use local databases, making the pipeline fully usable without internet access by using the local path to the database instead of a network link.

#### Integrating Uniref entries

To increase the versatility of ERMA and to address the CARD database’s limited focus on a set of ARGs, we included an optional automated retrieval routine for UniRef clusters via the /uniref/search endpoint. For that, we implemented a port of the UniProt REST API (https://rest.uniprot.org) in a custom script. The user selects the UniRef Cluster to retrieve from (100/90/50), the genes or functional targets to retrieve, and the maximum number of entries to retrieve per keyword. Returned entries are downloaded in FASTA format and automatically integrated into the CARD database prior to a subsequent similarity search, thus respecting UniProt rate limits by introducing delays and preserving API compliance. This optional function is intended to expand functional targets not present within the CARD set, increasing the versatility of the pipeline’s usage. However, this function does not replace a curated target database. If selected by the user, this analysis is performed and reported independently and therefore does not augment the main workflow. We also emphasize that the integrated entries are fundamentally different from the database itself and therefore constitute a hypothetical expansion to otherwise missing targets.

### Similarity search strategy

To identify the two parts of the fusion read, two separate similarity searches are performed for each read. EpicPCR fusion products typically consist of a fragment of the 16S rDNA gene linked to a fragment of an antibiotic resistance gene. Because these regions have different characteristics, with one representing a taxonomic marker and the other a protein-coding functional gene, they are analyzed using different similarity search strategies. Namely, the 16S rDNA fragment is analyzed at the nucleotide level, while the resistance gene fragment is analyzed at the protein level. To minimize processing time, we used DIAMOND BLASTX [27] with the default fast mode and USEARCH [26], using the local alignment mode for the similarity search. We selected these tools because of their balance of processing time and sensitivity. For both searches, the output is prefiltered with a configurable e-value threshold of 1 × 10^−5^. For the 16S rDNA gene search, a configurable minimum length of 200 nucleotides is required for a hit. This threshold is empirically determined and is intended to reduce processing time, while only slightly reducing sensitivity.

### Result integration and filtering logic

Merging and unifying the results of both similarity searches is a critical step in the pipeline. We use a custom script prioritizing specificity over sensitivity. Both similarity search results are parsed with Pandas [28]. In the absence of hits, a dummy line is generated, thereby increasing the pipeline’s robustness. In addition to the previously mentioned thresholds, several filtering steps are performed in the specified order:

1. A user-defined minimum percentage identity threshold is applied for every hit of both similarity searches to remove low-identity hits
2. The remaining hits are grouped by read identifier and ordered by descending percentage identity. Only the top hit per read identifier and similarity search is kept, while duplicates of that top hit are removed
3. The hit is only considered if it is present in both databases for the same read identifier

The top hits from both similarity searches are merged by the read identifier to produce the final result of both similarity searches for each read. This strategy assumes sufficient read quality and that the selected reference databases adequately represent the diversity of the respective taxonomic and functional sequence regions.

### Visualization

**Fig. 3:**
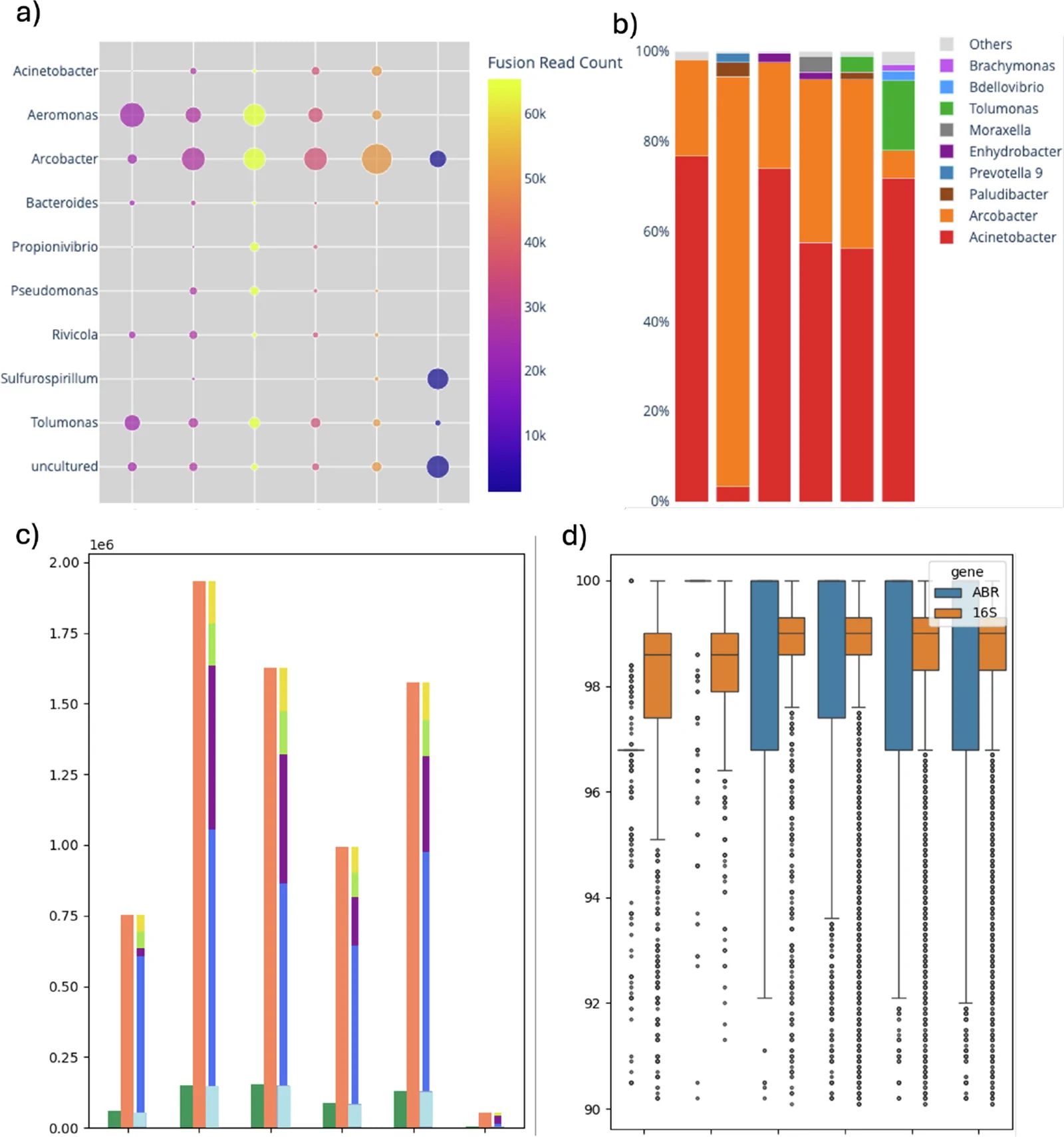
Example Panel of figures created with ERMA: **a** the bubble plot shows fusion reads of the top 10 abundant genera for the respective samples with their relative abundance shown by the size of the bubble and the total count of fusion reads shown by the bubble color (**b**) the stacked abundance plot shows the relative abundance of detected genera per sample (**c**) The attrition plot shows per sample the number of input FASTQ reads (darkgreen), the number of hits generated by the similarity searches (orange), the number of fusion reads retained after filtering (lightblue) and the number of hits removed from each filtering criterion (thin bar) displayed in a scientific notation (e.g., 1e6 corresponds to 10^6^ reads) (**d**) a boxplot of the percentage identity of the two different segments of the fusion reads per sample

The results are visualized using various plots that depict different levels of the processed reads. A bubble plot and a stacked abundance bar plot provide insight into the counts of filtered fusion reads per sample with the respective target and taxon at the genus level (Fig. 3a, b). Throughout the workflow, an overview table is generated iteratively with quantity metrics. Based on that, an attrition plot is created (Fig. 3c). It shows the number of input reads, generated hits through both similarity searches and filtered fusion reads, with reasons leading to filtering: hits with similarity below threshold, hits lower than the highest percentage identity per query, and hits only in one of the two databases. This enables comparability between runs and increases insight into the applied filtering. Furthermore, several boxplots are created (example: Fig. 3d) to depict the quality of the samples, namely the percentage identity, e-value, and alignment length, for each segment of the fusion read.

### Comparative evaluation

To assess the robustness and comparability of the pipeline, we selected five publicly available epicPCR datasets [9–13] and ran the generated reads through our pipeline using the same SILVA database as the original authors. The datasets were selected with a focus on the availability of sequence data and the completeness of abundance data, particularly with respect to taxonomic information. Many publications were excluded because genus-level data was unavailable, sequence files were missing, or the study fell outside the scope of ERMA, e.g., viral targets or unpublished databases (Table 2). All analyzes were executed on a dedicated Linux server running Ubuntu 20.04.6 LTS (Focal Fossa). The system was equipped with two Intel Xeon Silver 4216 processors (Cascade Lake) operating at a base frequency of 2.10 GHz. Each processor provided 16 physical cores with simultaneous multithreading, yielding 64 logical CPUs and 192 GB of RAM.

**Table 2:** EpicPCR-related publications assessed for reanalysis with ERMA

Year	First author	Sample origin	Data available	Re-analyzed	Comments
18	J. Cairns [33]	MOCK Community	yes	no	Unpublished database used
18	J. Hultman [9]	Two WWTPs Finland (inf./eff.)	yes	yes	Comparison on family level
19	H. Qin [34]	Sediment lake water	no	no	target unfit for pipeline
19	J. Florenza [35]	-	no	no	
20	L. Karnola*	Soil/manure	no	no	No genus data
20	M. Maunula*	Manure/manure fertilized soils	no	no	No genus data
20	A. Sarekoski*	WW - TP(inf./eff.)/hospital/rural	no	no	No genus data
20	E. G Sakowski [36]	Surface river	yes	no	target unfit for pipeline
21	Z. Wei [37]	5 urban WWTP	yes	no	No genus data
21	V. Roman [38]	River water/sediment	yes	no	
22	Z. Wei [39]	Agricultural soil	yes	no	No genus data
23	S. Dai [11]	Swine manure	yes	yes	
23	C. Knecht [40]	Wetlands treating municipal WW	yes	no	No ARG data
24	E. G. Lou [12]	WWTP (inf./eff.), secondary eff.	yes	yes	
24	S. Dekić Rozman [10]	2 WWTPs (inf./eff.)	yes	yes	
25	S. Liu [13]	Anaerobic digestion reactors	yes	yes	
25	V. Partanen [41]	Artificial bacterial community	yes	no	barcodes unfit to compare

Abbreviations: inf.—influent; eff.—effluent; WWTP—wastewater treatment plant. *Masterthesis

As verified ground truth is not available for any of these datasets, the concordance with published results was used as a relative performance measure. We decided to make comparisons at the lowest possible taxonomic level, comparing our results for an overlapping genus-ARG relation with those of the authors’ analysis. For consistency with the original analyses, we applied the same replicate-based host validation criteria reported by each study; while most datasets required detection in all three replicates to accept a host, Lou et al. accepted hosts detected in at least two of three replicates.

Sequencing data was downloaded using the SRA toolkit’s prefetch (https://github.com/ncbi/sra-tools) and converted into a paired-end configuration with fastq-dump. The file names were standardized with the corresponding sample names defined in the SRA Run Selector using a custom script. Whenever available, the version of the database specified by the authors was used. After analyzing the files with ERMA, we determined the runtime and subsequently used custom scripts to standardize the result format to match the analysis results reported in the respective publication.

We defined matches as taxa found in our results and by the authors, and mismatches as taxa found only by the authors and not by us. Taxa detected exclusively by ERMA were not included in the mismatch calculation. We adopted this approach to reflect the lack of ground-truth information and thereby avoided penalizing potentially valid ERMA findings.

To assess the potential impact of sequencing-mode-specific and similarity-search filtering parameters, additional sensitivity analyses were performed. One ONT dataset (Liu et al.) was reanalyzed using the DIAMOND –long-reads option, and the resulting genus-level concordance was compared with the original analysis. In a separate analysis, the DIAMOND e-value criterion was replaced by a bitscore criterion, and the resulting top hits were compared with those obtained using the original filtering strategy.

### Mock community validation experiment

To complement the concordance analysis of public datasets, we performed a controlled spike-in experiment using a defined mock community. The community consisted of five clinically relevant strains with known resistance genes: *Escherichia coli* (OXA-48), *Klebsiella pneumoniae* (OXA-48), *Klebsiella oxytoca* (OXA-48), *Serratia marcescens* (OXA-48), and *Acinetobacter baumannii* (OXA-23). Strains were cultivated separately at the Institute of Medical Microbiology (University Hospital Essen). Bacterial concentrations were adjusted to a McFarland standard of 1.0 (approximately 3 × 10^8^ cells/ml). Wastewater samples were collected from Ruhrlandklinik (University Hospital Essen, Germany; 229-bed facility) and processed immediately. The mock community was added in equal proportions (1:1:1:1:1) at four total concentrations in duplicate: 5 × 10^7^ (A), 1 × 10^7^ (B), 5 × 10^6^ (C), and 1 × 10^6^ (D) cells per sample. EpicPCR was performed using established primer (Table S1) and following established epicPCR protocols ([8–10]) with minor modifications and without applying a blocking PCR (see Protocol S1). Libraries were sequenced using ONT (v10 chemistry). The data was processed with ERMA using the default parameters. In parallel, the same spiked-in wastewater sample was subjected to standard 16S rDNA gene amplicon sequencing. 16S rDNA reads from that approach were analyzed using USEARCH against the SILVA database with filtering criteria analogous to those implemented in ERMA. The overlap between genera detected by ERMA and the 16S workflow was determined per sample. To account for the intrinsic asymmetry between epicPCR, which represents the ARG-positive fraction, and 16S profiling, which captures the broader microbial community, genera detected exclusively by 16S were not considered mismatches. Overlap was therefore calculated as the proportion of ERMA-detected genera that were also detected by the standalone 16S workflow.

## Results

Using ERMA, we successfully processed five heterogeneous public epicPCR datasets comprising 4–8 samples (with three replicates, each) and 0.29–0.97 Gb input bases per study. As a result, we generated interpretable and comparable fusion read outputs across Illumina and ONT sequencing platforms.

The reported wall-clock time was obtained under low system load and ranged from 84.4 to 245.3 min (Table 3). The USEARCH similarity search was the most dominant runtime contributor, accounting for over 90% of CPU time in every run (data not shown).

**Table 3:** Results from the epicPCR concordance analysis with ERMA, showing the sequencing platform employed for generating the data, number of samples and replicates, input FASTQ reads and bases (in Gigabases) and the calculated runtime. For all datasets, the analysis was run on 64 cores

First author	Sequencing platform	#samples/#replicates	Input Reads	Input Bases	Wallclock time [m]
S. Liu	ONT	8/3	5.7 × 10^5^	0.29 Gb	93.2
J. Hultman	Illumina	4/3	7.0 × 10^5^	0.31 Gb	84.5
S. Dai	Illumina	4/3	1.3 × 10^6^	0.35 Gb	84.4
E. G. Lou	ONT	6/3	4.3 × 10^5^	0.38 Gb	99.0
S. Dekić Rozman	Illumina	7/3	2.5 × 10^6^	0.97 Gb	245.3

### Concordance analysis

The procedure for comparing the general metrics of the five datasets was similar to creating an attrition plot (Visualization section) but pooled by publication rather than by sample. For all five datasets, the number of input reads ranged from 4.3 × 10^5^ reads (Lou et al.) to 2.5 × 10^6^ reads (Dekić Rozman et al.) (Table 3). The number of output fusion reads ranged between 1.0 × 10^5^ reads (Lou et al.) and 6.8 × 10^5^ reads (Dekić Rozman et al.). Across datasets, the proportion of input reads not retained as filtered fusion reads ranged from 1.06% to 79.56%. Among the reads removed during filtering, the predominant filtering reason varied between datasets and was either sub-threshold DIAMOND alignments (61.22% in Dai and 59.87% in Hultman et al.), keeping only the DIAMOND hits with the highest percentage identity per read identifier, therefore excluding remaining hits (79.96% in Dekić Rozman et al.), or queries matching only one instead of two databases (88.69% in Liu). These differences highlight dataset-specific bottlenecks at different stages of similarity integration (Fig. S1).

To compare the results obtained with ERMA with those reported in the publications, we counted the genera that matched both approaches as matches and those present in the authors’ analysis but absent from ERMA as mismatches. The dataset of Hultman et al. was the only dataset tested at the family level, while the others were analyzed at the genus level. The results of the match analyzes are depicted as bar plots per sample, with the percentage of matches as positive values and the percentage of mismatches as negative values (Table 4; Table nus assignments for all 722 evaluated top hits (TabS2; Fig. S2).

Additional sensitivity analyses showed no change in the observed concordance when the ONT dataset of Liu et al. was reanalyzed using DIAMOND’s –long-reads option (Table S3). Similarly, replacing the e-value criterion with bitscore-based filtering resulted in identical query ID, AMR gene family, and genus assignments for all 722 evaluated top hits (Table S4) .

**Table 4:** Results of the average matching/mismatching rates for the five publications reanalyzed with ERMA

First author	# Genera	SILVA v.	M/MM ratio	Comments
S. Dai	19	138.1	78.95	
S. Dekić Rozman	167	138.1	83.32	
J. Hultman	78 (families)	128	70.67	
S. Liu	10	138.1	78.75	
E. G. Lou	452	138	83.04	hosts accepted if detected in ≥2 of 3 replicates, following the original study

### Validation using a defined mock community

When using ERMA for analyzing wastewater samples spiked in with a defined community, the expected mock genera could be recovered by epicPCR followed by ERMA analysis at all tested concentrations. The number of fusion reads assigned to each mock genus decreased proportionally with the spike-in concentration, consistent with the expected concentration relationship (Fig. S4). Notably, the OXA-48-negative genus *Acinetobacter* exhibited a comparable decrease across concentrations, despite not carrying the targeted OXA-48 marker. This pattern suggests low-level mislinkage events, possibly due to the absence of a blocking PCR step. In epicPCR workflows, insufficient suppression of intermediate amplicons has been reported to increase the probability of artificial gene-host associations, which may explain the observed signal for *Acinetobacter*.

Comparing the epicPCR results with those obtained through standalone 16S rDNA profiling of the same spiked-in wastewater sample revealed substantial overlap between the two approaches. Across eight samples (A1–D2), genus-level concordance ranged from 81.48% to 90.0%, with a mean overlap of 84.96% (Fig. S3). Because standalone 16S profiling captures the total microbial community whereas ERMA detects only the ARG-positive fraction, genera detected exclusively by 16S were not considered discordant. Among the genera detected by ERMA, only 2–5 per sample were not recovered by standalone 16S profiling. These ERMA-only detections were predominantly attributable to three recurrent taxa: *Mangrovibacter*, *Kosakonia*, and *Salmonella*. Given that epicPCR and standalone 16S sequencing targeted different 16S regions and primer sets, these recurrent discrepancies likely reflect primer bias or taxonomic resolution differences within *Enterobacterales* rather than systematic errors in the ERMA workflow.

## Discussion

EpicPCR datasets rarely provide a validated reference set of true gene-host associations, making formal ground-truth benchmarking challenging. Although validation strategies such as synthetic polyacrylamide bead controls [8] or defined mock communities [13] have demonstrated validation in principle, such controls are not routinely included in publicly available datasets. In this context, cross-study concordance analysis represents the most informative strategy for evaluating analytical consistency and robustness in heterogeneous epicPCR experiments. This limitation also applies to the present concordance analysis, in which ERMA was evaluated against author-reported results derived from heterogeneous, often unpublished, custom workflows.

ERMA achieved substantial concordance across all five publicly available datasets, with an average concordance ranging from 70% to 83%. These values are notable given the diversity of sample origins, sequencing platforms employed, and analytical strategies used in the original studies. Concordance rates were comparable for datasets generated using either Illumina or ONT sequencing. The observed agreement suggests that ERMA reliably reproduces the dominant taxonomic signals reported by the original authors, even without dataset-specific tuning.

Even within a single epicPCR study, replicate quality can vary due to methodological factors such as droplet generation efficiency, amplification bias, or sequencing depth. Because such variation may not be apparent from results reported at the sample level, quality control at the replicate level can be important when assessing the reliability of individual results. ERMA provides quality control summaries and attrition plots that make differences in sequencing depth, filtering, and fusion-read recovery visible, thereby helping users identify potentially problematic replicates and make informed decisions about their inclusion in downstream analyses.

Interpreting mismatches requires particular caution. In this concordance analysis, genera detected by ERMA but absent in the source publications were excluded from the mismatch calculation. This decision originates from the asymmetry of available information: the absence of a reported taxon in a publication does not constitute evidence that the taxon is a false positive by ERMA, especially given the wide variation in bioinformatic tools, filtering criteria, and reporting thresholds across studies. Therefore, excluding ERMA-only detections avoids penalizing the pipeline for potentially valid findings, allowing the concordance analysis to focus on the recovery of previously reported taxa. Although this approach likely underestimates the total true-positive signal captured by ERMA, it provides a conservative and transparent measure of concordance under realistic epicPCR conditions.

The attrition patterns observed within the datasets provide insight into both the technical challenges of epicPCR and the utility of a standardized analysis framework. A large fraction of reads were filtered during similarity search integration, mostly due to subthreshold alignments, best hits, or incomplete linkage between databases. These losses are expected given the complexity of epicPCR read fusion, which is prone to chimera formation, uneven amplification, and platform-specific error profiles. However, the large range of filtered input reads and differences in filtering reasons underline the heterogeneity of the datasets. The larger input size of the Dekić Rozman et al. [10] dataset led to correspondingly higher absolute numbers of retained fusion reads but did not substantially alter the relative structure of attrition, underscoring the pipeline’s scalability.

To complement the concordance analysis of public datasets, we performed a controlled spike-in experiment using a defined mock community in wastewater. In this setting, all expected genera were consistently recovered by ERMA. Genus-level overlap with independent 16S profiling exceeded 80% in all replicates and remained stable across decreasing input concentrations. The small number of recurrent non-overlapping genera is plausibly explained by primer and amplicon-region differences rather than systematic misclassification. This experiment supports the robustness of ERMA’s taxonomic integration and filtering logic under controlled conditions.

A key feature of ERMA is its emphasis on reproducibility and harmonization of data analysis. By providing an end-to-end, version-controlled Snakemake-based workflow with minimal required user input, ERMA reduces the analytical degrees of freedom to increase comparability across epicPCR studies. The modular handling of database preparation and the dynamic integration of specific UniRef entries allow users to adapt the pipeline to specific research questions, thereby increasing the flexibility to target beyond AMR. Those aspects of flexibility and standardization are particularly important in a field where methodological diversity has thus far limited cross-study comparability.

Several limitations should be acknowledged. First, as discussed above, the absence of validated ground-truth datasets for epicPCR constrains objective performance assessment. Although concordance with published results provides a pragmatic evaluation strategy, we increased confidence by additionally including a validation step based on a characterized mock community. Future benchmarking efforts would benefit from more studies employing standardized mock communities or synthetic fusion reads with defined host-gene associations. This approach could increase the robustness of the pipeline and therefore benefit the field of epicPCR data analysis. In addition, ERMA’s general performance depends on the completeness, curation, and taxonomic resolution of the selected reference databases. The deliberate prioritization of specificity in the filtering logic, particularly the retention of only the highest-identity hit present in both databases, may exclude borderline but biologically relevant associations. This conservative design is chosen to reduce false-positive linkage while aiming for computational speed. In addition, the current workflow assumes sufficient read quality for reliable similarity searches. Future versions could incorporate dedicated read-quality filtering or error-correction steps during preprocessing to reduce the influence of low-quality reads, particularly for long-read datasets. In the future, these limitations could be addressed through a systematic comparison of alternative filtering strategies and the incorporation of improved long-read error correction approaches. Similarly, although ERMA currently uses USEARCH for 16S similarity searches, open-source alternatives such as VSEARCH [42] could provide a community-maintained alternative. Future versions of ERMA could include VSEARCH as an alternative similarity searching tool, although this would require an initial separation step of the fusion reads. As reference databases and experimental standards mature, ERMA provides a modular framework that can be extended and refined to support increasingly standardized and comparable epicPCR analyzes.

## Conclusion

In this study, we introduce ERMA, an open-source, reproducible, scalable, and versatile Snakemake-based pipeline that supports the standardized analysis of epicPCR data across sequencing platforms and experimental designs, facilitating the consistent and comparable implementation of epicPCR workflows. The explicit handling of attrition and filtering decisions further improves interpretability as well as comparability across datasets. Evaluation of five publicly available datasets showed that, despite the absence of a definitive ground truth, the pipeline consistently reproduces the majority of taxonomic signals reported in the original studies. An additional strength of this work lies in the inclusion of validation using a defined mock community, providing an independent benchmark to assess taxonomic recovery accuracy under controlled conditions.

ERMA provides a practical step toward harmonizing the analysis of epicPCR data. Its flexible design and emphasis on reproducibility make it well-suited for reanalysis of existing data and for future studies that aim to compare epicPCR results across experiments, laboratories, and applications.

## Supplementary information

Below is the link to the electronic supplementary material.

Supplementary Information

## Data Availability

All epicPCR sequences generated for this study are available in the European Nucleotide Archive (ENA) under the accession number PRJEB124414.

## Code availability

Project name: ERMA. Project home page: https://github.com/IKIM-Essen/ERMA/releases/tag/v1.0.0. Operating system(s): Linux. Programming language: Snakemake, Python. Other requirements: Conda/Mamba. License: MIT license.

## Abbreviations

AMR: Antimicrobial Resistance
ARG/ABR: Antibiotic resistance gene
BLAST: Basic Local Alignment Search Tool
bp: Base pairs
dsDNA: Double-stranded DNA
eff.: effluent
epicPCR: Emulsion, Paired Isolation and Concatenation PCR
ERMA: EpicPCR Resistome-Microbiome Analyzer
Gb: Gigabases
inf.: influent
ONT: Oxford Nanopore Technologies
PCR: Polymerase Chain Reaction
qPCR: Quantitative PCR
WW: Wastewater
