# Supplementary material for: ERMA: a harmonizing epicPCR data analysis tool
Source: BMC Genomics. 2026 Oct 9;27:842. doi: 10.1186/s12864-026-13418-y (PMC13660262; doi:10.1186/s12864-026-13418-y)
Supplement: Supplementary file 1 [file 12864_2026_13418_MOESM1_ESM.pdf]

13 Electronic supplementary material

Supplementary Protocol S1: epicPCR Workflow for Mock Validation

1. Mock community preparation

   Individual bacterial strains were cultured separately in liquid medium. Cell density was adjusted to McFarland 1.0 (3×10^8 cells/mL). Strains were combined in equal proportions. The mock mixture was spiked into freshly collected clinical wastewater at defined cell numbers (1×10^6–5×10^7 total cells).

2. Polyacrylamide bead encapsulation

   Cells were embedded in polyacrylamide microbeads using an emulsion-based polymerization approach. Acrylamide/BIS solution and ammonium persulfate were mixed with the cell suspension. Emulsification was performed in mineral oil containing Span80, Tween80, and Triton X-100. Polymerization was initiated by addition of TEMED and incubated at room temperature (∼90 min). Beads were recovered by organic extraction and washed repeatedly to remove residual oil. Beads were resuspended in Tris–KCl buffer and filtered through a 35 µm mesh. Single-cell occupancy was assessed by DAPI staining and fluorescence microscopy.

3. Fusion PCR (Linking AMR and 16S Fragments)

   Beads were re-emulsified in ABIL oil. PCR mix included: OXA-48-specific forward primer, 16S primer with linker, OXA-48 linker primer, GC buffer, MgCl2, dNTPs, high-fidelity polymerase, Thermocycling: Initial denaturation 33 cycles

(denaturation–annealing–extension) Final extension Emulsions were pooled after amplification.

4. Post-PCR Emulsion Breaking and Cleanup

   Emulsions were disrupted using water-saturated diethyl ether. Ethyl acetate wash performed. Residual solvents evaporated. DNA purified using AMPure XP magnetic beads.

5. Nested PCR

   Short amplicon (∼500 bp) Targeted genus-level 16S region. 40 PCR cycles. The reaction used high-fidelity polymerase and blocking primers to reduce chimera formation.

6. Quality Control

   DNA quantified using fluorometric dsDNA assay. Fragment size verified using TapeStation (high-sensitivity D1000 kit).

7. ONT Library Preparation and Sequencing

   DNA repair and end-prep performed. Native barcodes ligated. Samples pooled and purified (AMPure XP). Sequencing adapters ligated. Library loaded onto ONT flow cell (Native Barcoding Kit V14). Sequencing performed according to manufacturer instructions.

Table S1 Primer used for epicPCR mock community experiment.

Name	Description	Oligonucleotide Sequence	Reference
OXA-48 F1	OXA-48 fusion Primer	5´-AATTTGGCGGGCAAATTC-3´	[10]
OXA-48 R3	OXA-48 nested Primer	5´-GTAAMRATGCTTGGTTCGC-3´	[43]
pH’	pH’ 16S Primer	5´-AAGGAGGTGATCCAGCCGCA-3´	[44]
785R	785R 16S Primer	5´-GACTACHVGGGTATCTAATCC-3´	[45]
OXA-48 linker	OXA-48 linker Primer	5´-GWATTACCGCGGCKGCTG TGTTTTTGGTGGCATCGAT-3´	[10, 43]
1492R	1492R 16S Primer	5´-GGTTAACCTTGTTACGACTT-3´	[8]

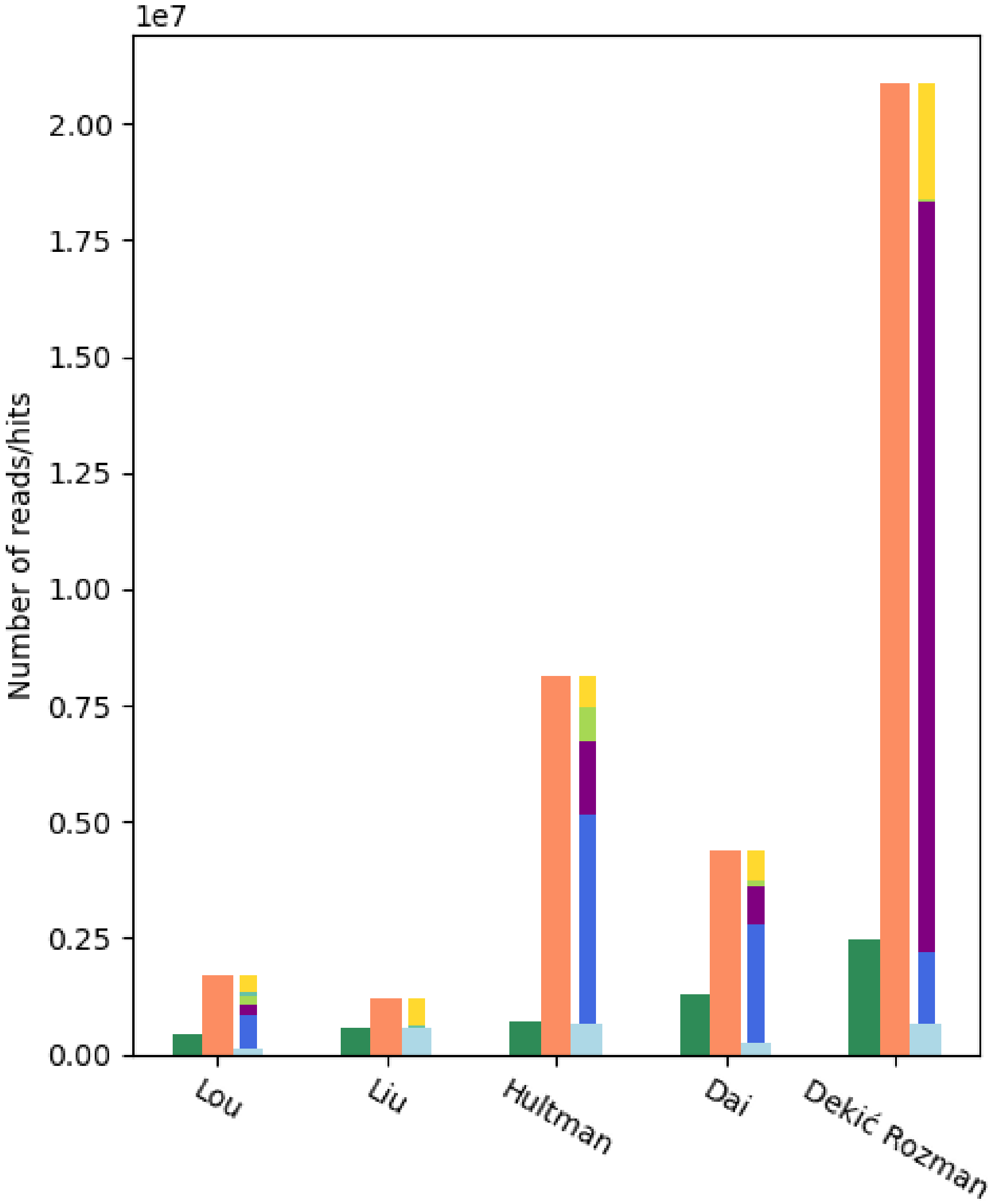

Fig. S1 ERMA attrition plot of the pooled samples of every used publication dataset. The plot shows four bars per dataset (from left to right): total number of input reads (dark green), number of hits generated for this dataset with both similarity searches (orange), and number of filtered true fusion reads (light blue). The thin bar indicates the filtered hits with respective reasoning (from bottom to top): DIAMOND hits with percentage identities under the threshold (dark blue), DIAMOND hits not being the top hit per read identity window (purple), USEARCH hits with percentage identities under the threshold (light green), USEARCH hits not being the top hit per read identity window (cyan) and hits only from one of the databases (yellow).

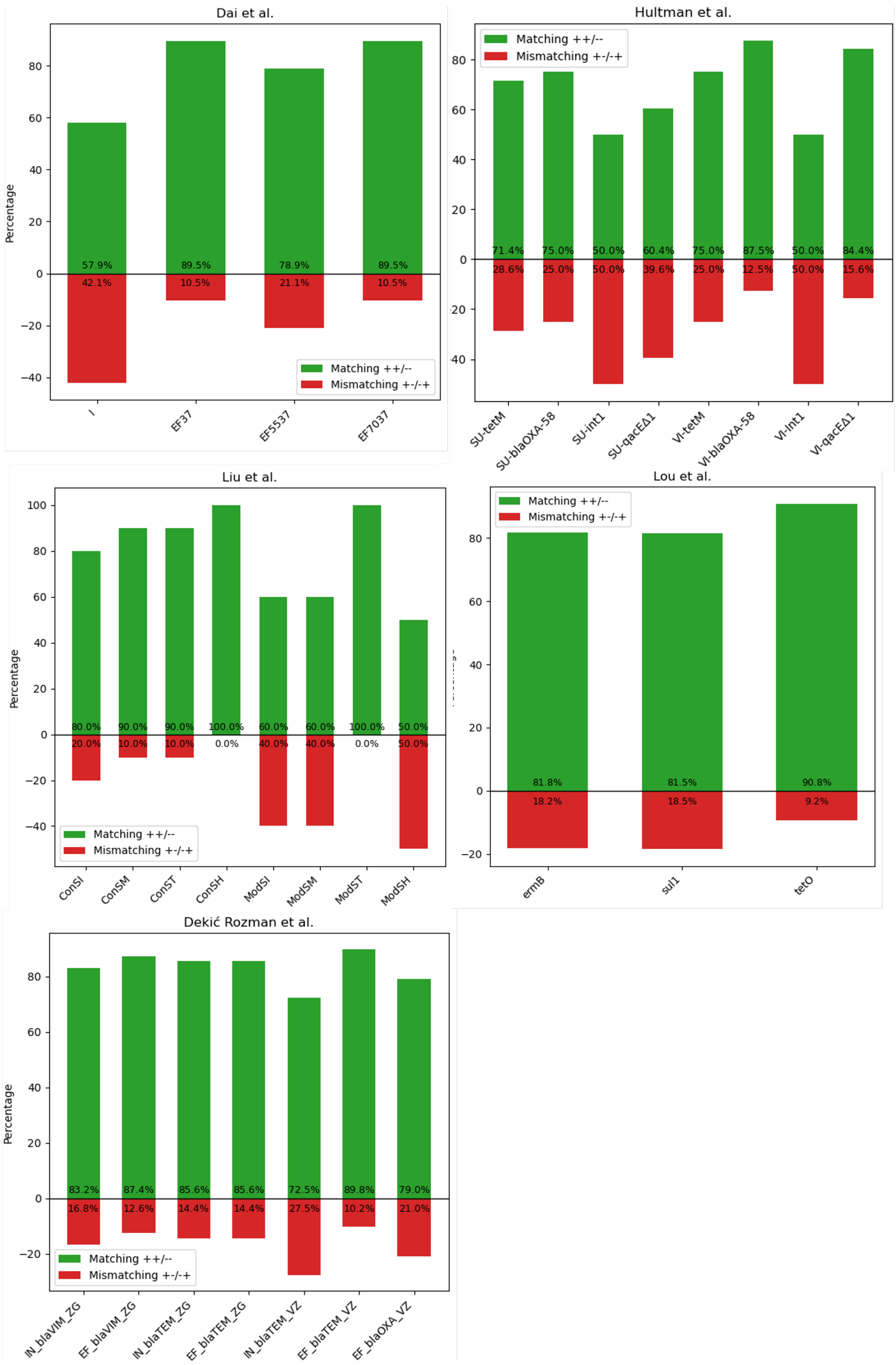

Fig. S2 Match/Mismatch bar plots of the re-analysis of five publicly available datasets with ERMA. The relative matching rate is depicted as a positive bar (green), and the mismatch rate is depicted as a negative bar (red).

Table S2 Match/Mismatch statistics of all analyzed datasets with the samples and genera from the respective dataset with the match/mismatches found with our analysis.

First Author	sample	genus count	match count	mismatch count	percentage match count	percentage mismatch count
Dai et al.	I	19	11	8	57.9	42.1
	EF37	19	17	2	89.5	10.5
	EF5537	19	15	4	78.9	21.1
	EF7037	19	17	2	89.5	10.5
Hultman et al.	SU-tetM	28	20	8	71.4	28.6
	SU-blaOXA-58	12	9	3	75	25
	SU-int1	10	5	5	50	50
	SU-qacEΔ1	48	30	18	62.5	37.5
	VI-tetM	16	11	5	68.8	31.2
	VI-blaOXA-58	8	7	1	87.5	12.5
	VI-Int1	22	11	11	50	50
	VI-qacEΔ1	64	54	10	84.4	15.6
Liu et al.	ConSI	10	8	2	80	20
	ConSM	10	9	1	90	10
	ConST	10	9	1	90	10
	ConSH	10	10	0	100	0
	ModSI	10	6	4	60	40
	ModSM	10	6	4	60	40
	ModST	10	10	0	100	0
	ModSH	10	5	5	50	50
Lou et al.	ermB	44	36	8	81.8	18.2
	sul1	233	189	44	81.1	18.9
	tetO	65	59	6	90.8	9.2
Dekic Rozman et al.	IN_blaVIM_ZG	167	139	28	83.2	16.8
	EF_blaVIM_ZG	167	146	21	87.4	12.6
	IN_blaTEM_ZG	167	143	24	85.6	14.4
	EF_blaTEM_ZG	167	143	24	85.6	14.4
	IN_blaTEM_VZ	167	121	46	72.5	27.5
	EF_blaTEM_VZ	167	150	17	89.8	10.2
	EF_blaOXA_VZ	167	132	35	79	21

Table S3 Differences of comparison for the liu dataset with (lr) and without (nlr) the option ”–long-reads” within the similarity search with USEARCH.

**sample**	**genus count**	**match count (nlr)**	**mismatch count (nlr)**	**match count (lr)**	**mismatch count (lr)**
ConSI	10	8	2	8	2
ConSM	10	9	1	9	1
ConST	10	9	1	9	1
ConSH	10	10	0	10	0
ModSI	10	6	4	6	4
ModSM	10	6	4	6	4
ModST	10	10	0	10	0
ModSH	10	5	5	5	5

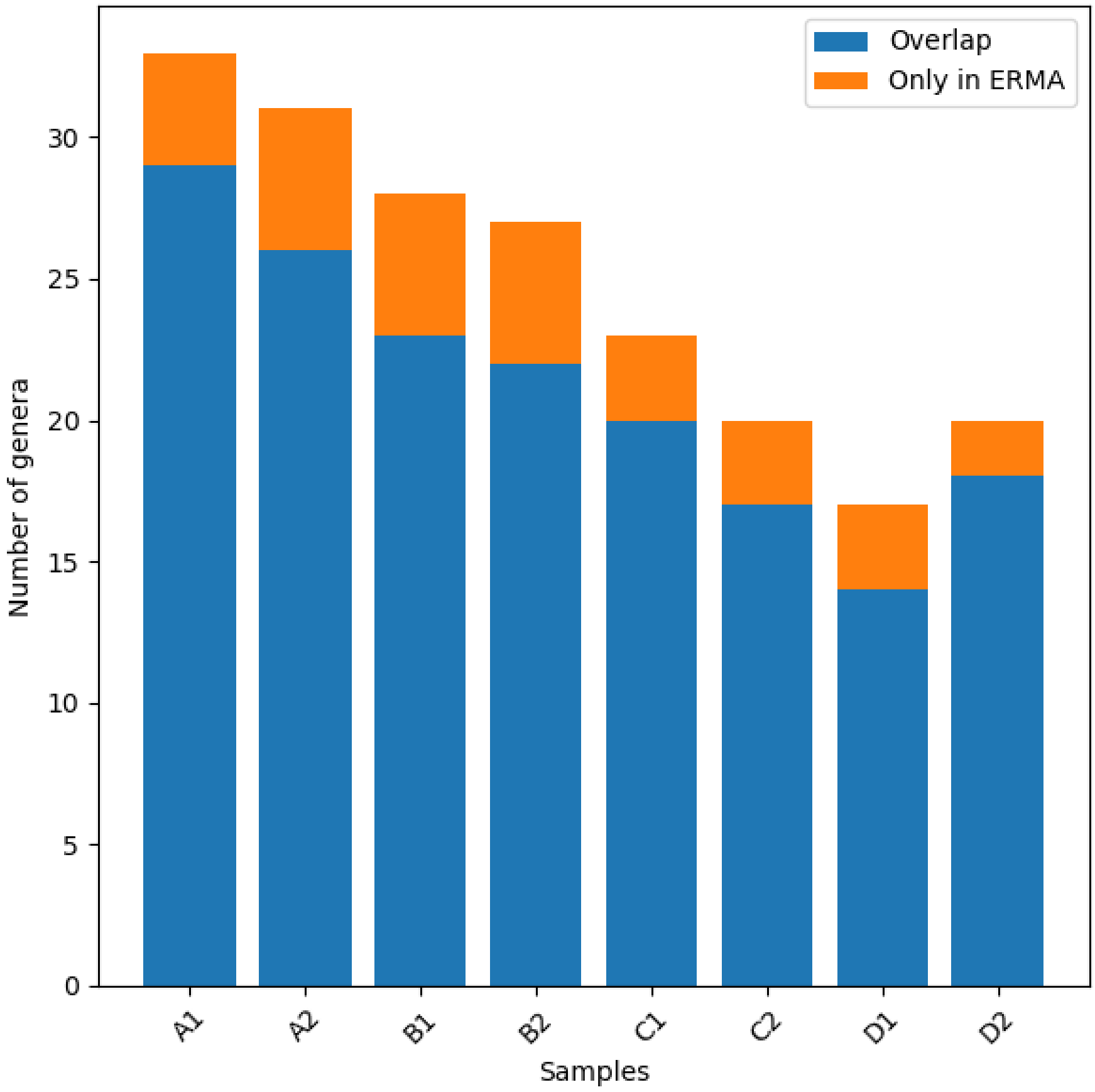

Fig. S3 Comparison of genus-level overlap between ERMA analysis of mock-spiked wastewater samples and standalone 16S rDNA gene analysis of the corresponding wastewater sample. Bars represent the total number of genera detected by ERMA at different spike-in concentrations (5×10^7 (A), 1×10^7 (B), 5×10^6 (C) and 1×10^6 (D) cells per sample in duplicates 1/2). The genera detected by both approaches are shown in blue, while genera detected exclusively by ERMA are shown in orange.

Table S4 Differences of comparison for the filtering of the test datasets with Evalue vs. bitscore.

	**Evalue vs. bitscore**	**percentage**
Number of records	722	
Query ID identical	722	100%
AMR Gene Family identical	722	100%
Genus identical	722	100%
Records with any discrepancy	0	

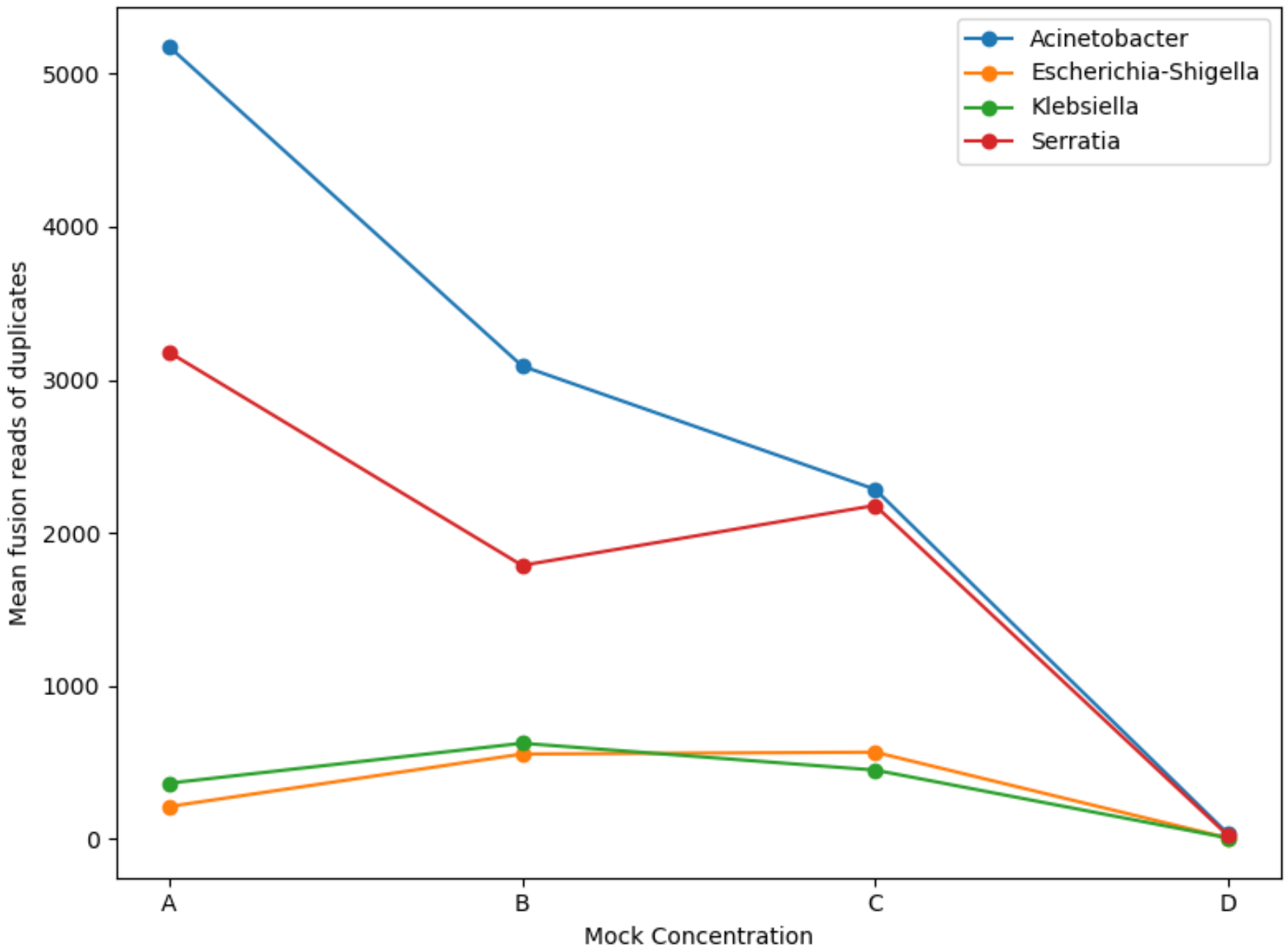

Fig. S4 Detected number of fusion reads of the mock community samples with the concentrations: $5{\times}10^7$ (A), $1{\times}10^7$ (B), $5{\times}10^6$ (C) and $1{\times}10^6$ (D) cells per sample.
